# Radiomics-based high-resolution CT analysis for differentiating primary tumor sources of pulmonary metastases

**DOI:** 10.3389/fonc.2026.1908570

**Published:** 2026-07-23

**Authors:** Kangning Liu, Yingnan Zhang, Xiaoxuan Xie, Na Li, Genyun Liu, Weijia Li, Yuhang Wu, Bowen Hu, Fei Zhao, Zhiling Wan, Yifan Zhang, Yun Zhou, Xiaojin Wu

**Affiliations:** 1 Xuzhou Medical University, Xuzhou, Jiangsu, China; 2 Xuzhou Clinical School of Xuzhou Medical University, Xuzhou, Jiangsu, China; 3 Xuzhou Central Hospital Affiliated to Medical School of Southeast University, Xuzhou, Jiangsu, China; 4 The Affiliated Xuzhou Municipal Hospital of Xuzhou Medical University, Xuzhou, Jiangsu, China; 5 The First People’s Hospital of Xuzhou, Xuzhou, Jiangsu, China

**Keywords:** high-resolution CT, logistic regression, machine learning, pulmonary metastases, radiomics

## Abstract

**Objective:**

To evaluate machine learning models based on HRCT radiomic features for distinguishing breast and colorectal cancer pulmonary metastases, and interpret the optimal model to aid clinical decision-making.

**Methods:**

This retrospective study enrolled 85 patients with pathologically confirmed pulmonary metastases. After radiomic feature extraction, the cohort was divided into a training set (n=59) and an independent test set (n=26) at a 7:3 ratio via stratified sampling. Data were processed with Z-score normalization, variance thresholding and PCA (45 principal components). Five classifiers were constructed: LR, linear SVM, RF, XGBoost and LightGBM. Model stability and performance were assessed by 5-fold stratified cross-validation and independent test validation.

**Results:**

The 45 principal components accounted for 99.92% of cumulative variance. LR showed optimal performance, with a test AUC of 0.9821, classification accuracy of 84.62%, and a mean cross-validation AUC of 0.9606 (95% CI: 0.8887–0.9895). The small training-test AUC difference (0.0179) indicated no severe overfitting. SVM ranked second (test AUC = 0.9405), while XGBoost and RF exhibited significant overfitting and LightGBM underfitting. The model’s decision relied on key texture features; only GLSZM non-uniformity differed significantly between groups, consistent with their pathophysiological characteristics.

**Conclusion:**

The PCA-reduced and regularization-optimized LR model has excellent generalization, stability and clinical interpretability in differentiating pulmonary metastases from breast and colorectal cancers. This study preliminarily highlights the potential of LR in high-dimensional small-sample scenarios, and provides a foundational methodological reference for future large-scale multicenter diagnostic research.

## 1 Introduction

The lung is a highly susceptible target organ for malignant tumor metastasis, as its unique anatomical and physiological characteristics create favorable conditions for cancer cell colonization. Hemodynamically, the pulmonary circulation occupies a central position within the systemic circulation, characterized by a slow blood flow velocity and high local coagulation-fibrinolysis system activity. These properties facilitate the retention and subsequent metastasis formation of circulating tumor cells within the pulmonary microvasculature. Structurally, the lung not only possesses a dense capillary network but also an abundant distribution of lymphatic tissues. This dual-filtration mechanism ensures that all systemic venous return undergoes meticulous screening in the lungs. Consequently, during hematogenous metastasis of malignant tumors, the lung frequently emerges as the second most common metastatic site, following the liver.

Pulmonary metastases generally retain the biological hallmarks of their primary tumors. Although high-resolution CT (HRCT) provides superior clarity of lesion margins and internal structures compared to conventional non-contrast scans, certain subtle features remain indistinguishable to the naked eye. The ability to infer the primary tumor origin based on imaging features would significantly enhance clinical diagnostic efficiency, particularly for metastatic lung cancers with an unknown primary site. While pathological biopsy remains the gold standard for diagnosis, its invasiveness limits its routine clinical application.

Radiomics involves the high-throughput extraction of extensive quantitative imaging features from medical images, utilizing these data to comprehensively characterize the macroscopic and microscopic morphological and functional traits of tumors (1, 2). Currently, radiomics has been successfully applied to differentiate benign from malignant pulmonary nodules (3, 4) and primary from metastatic lung tumors (5). A recent CT-based radiomics study achieved excellent performance in preoperative prediction of lymph node metastasis in pancreatic cancer, with an AUC ranging from 0.86 to 0.94, demonstrating its reliable value in noninvasive tumor evaluation (6). However, studies focusing on differentiating the specific origins of primary tumors remain scarce. A recent retrospective study developed a multi-classifier radiomics model for identifying primary origins of pulmonary metastases, yet direct comparative evidence between breast and colorectal cancer metastases remains limited (7). The deep integration of machine learning algorithms with radiomic features has unveiled vast application prospects in non-invasive diagnosis, prognostic evaluation, and gene mutation prediction of solid tumors (8–11).

The radiomic signatures of pulmonary metastases can effectively reflect the distinct pathophysiological properties of different primary tumors, primarily manifesting as deep texture and morphological differences in medical images (12–14). In clinical practice, breast cancer and colorectal cancer are among the most common primary sources of lung metastases. They exhibit pronounced biological differences in their tumor microenvironments, angiogenesis mechanisms, and stromal infiltration patterns (15–17). Traditional clinical diagnosis heavily relies on invasive core needle biopsies. However, this technique has notable limitations, causing considerable patient discomfort and carrying risks of potential complications. Moreover, due to spatial tumor heterogeneity, localized sampling often fails to accurately capture the global characteristics of the lesion (18–20).

Addressing this clinical challenge, our study innovatively utilizes HRCT radiomic features and compares the performance of multiple machine learning models. We focus on selecting the optimal model with superior generalization and stability. By employing a multi-layered interpretability analysis framework to elucidate its decision-making mechanisms, this study aims to provide a reliable and interpretable novel non-invasive diagnostic approach for clinical practice.

## 2 Materials and methods

### 2.1 Study population

Clinical and imaging data of patients with pathologically confirmed pulmonary metastases who underwent HRCT examinations at Xuzhou Central Hospital between January 2019 and December 2024 were retrospectively collected.

Inclusion criteria: (1) Definitive pathological diagnosis obtained via surgery or biopsy; (2) Chest HRCT scan performed within 2 weeks prior to surgery; (3) Clear, identifiable CT images without motion artifacts or other factors compromising diagnostic quality.
Exclusion criteria: (1) History of invasive thoracic procedures, such as lung puncture or ablation; (2) Incomplete clinical or pathological data.

A total of 85 patients were included in this study, comprising 38 patients with breast cancer and 47 with colorectal cancer. Sample size estimation: Assuming an expected AUC = 0.85, setting the primary tumor source ratio based on actual enrollment (breast cancer: colorectal cancer ≈ 0.9:1), α = 0.05, and statistical power = 0.80, the Hanley & McNeil method estimated a minimum required positive sample size of 32 cases and a total sample size of at least 64 cases. The 85 cases included in this study met the power requirements. The baseline clinical characteristics of the enrolled patients are summarized in **Table 1**. Briefly, the colorectal cancer group consisted of 47 patients (34 males, 13 females) with a mean age of 66.6 ± 11.2 years (range: 36–86 years). The breast cancer group consisted of 38 patients (all females) with a mean age of 58.0 ± 10.1 years (range: 39–73 years). The age difference between the two groups was statistically significant (t = 3.68, P < 0.001). All patients were at stage IV (M1) disease. No patient had received local interventional therapy for pulmonary lesions prior to HRCT examination. This study was approved by the Medical Ethics Committee of our hospital. Due to its retrospective nature, informed consent from patients was waived.

**Table 1:** Baseline clinical characteristics of the study population.

Characteristic	Colorectal cancer(n = 47)	Breast cancer(n = 38)	Statistic	P-value
Age (years), mean ± SD	66.6000 ± 11.2000	58.0000 ± 10.1000	t = 3.6800	< 0.001
Sex, n (%)			χ² = 48.2000	< 0.001
Male	34 (72.3)	0 (0.0)	—	—
Female	13 (27.7)	38 (100.0)	—	—
Clinical stage, n (%)			—	—
IV	47 (100.0)	38 (100.0)	—	—
Prior local therapy, n (%)	0 (0.0)	0 (0.0)	—	—

Continuous variables are shown as mean ± SD; age and radiomic features were compared by t-test and Mann-Whitney U test, respectively. Categorical variables are n (%), compared by chi-square test. Two-sided P < 0.05 was considered significant.

### 2.2 CT image acquisition and reconstruction

Scans were performed using a multi-detector spiral CT scanner (SOMATOM Definition Flash, Siemens Healthcare). Scanning parameters: detector collimation width 64 mm × 0.6 mm or 128 mm × 0.6 mm, tube voltage 120 kV, with tube current modulated by an automatic exposure control system. Images were reconstructed with a slice thickness of 1.0–1.5 mm and an interval of 1.0–1.5 mm, without contrast agents. Image data were stored in DICOM format and imported into 3D Slicer software (v5.6.2) for region of interest (ROI) delineation of pulmonary metastases.

### 2.3 Radiomic feature extraction

ROI delineation was performed independently by two radiologists with over 5 years of diagnostic experience, blinded to the patients’ clinical information. A random subset of 30 cases was selected to calculate the inter-observer intraclass correlation coefficient (ICC). The mean ICC for shape features was 0.892 (range: 0.812–0.946), and for texture features was 0.864 (range: 0.785–0.923). The overall ICC exceeded 0.75, indicating excellent inter-observer agreement. Discrepancies between the two physicians were resolved through consensus discussion to determine the final ROI.

The “Radiomics” extension module in 3D Slicer was utilized to extract features, including first-order statistics, shape features, and texture features (Gray Level Co-occurrence Matrix [GLCM], Gray Level Run Length Matrix [GLRLM], Gray Level Size Zone Matrix [GLSZM], etc.), yielding a total of 130 features. Extraction parameters: gray-level quantization bin width = 25, texture feature distance factor = 1 mm, symmetrical GLCM (symmetricalGLCM=True). ROIs were delineated slice-by-slice in 3D, encompassing the solid tumor region while avoiding necrotic areas and normal lung tissue. For patients with multiple pulmonary metastatic lesions, only the largest solid lesion with well-defined borders and without macroscopic necrosis was selected as the target lesion for ROI delineation, in accordance with the measurable lesion criteria of RECIST 1.1. All analyses in this study were based on a single target lesion per patient to avoid intra-patient feature heterogeneity and ensure the consistency of feature extraction across the entire cohort. These high-dimensional features quantify the spatial arrangement of voxels and gray-level distribution differences within the tumor at a microscopic level, which have been widely validated by previous basic and clinical studies to tightly correlate with tumor cell density, microangiogenesis, and local necrosis extent (21–24).

All radiomic feature extraction pipelines in this study fully comply with the standardized mathematical definitions, discretization rules and 3D texture calculation protocols proposed by the Image Biomarker Standardization Initiative (IBSI) (25). The bin width of 25, 1 mm texture distance factor and symmetric GLCM setting adopted in our workflow strictly follow IBSI benchmark parameters, which eliminates systematic deviation caused by inconsistent software configurations and improves cross-study reproducibility of GLSZM, GLCM and GLRLM texture metrics.

### 2.4 Data preprocessing and dimensionality reduction

Feature data were output as CSV files. Data processing strictly adhered to the “split first, then preprocess” protocol, where all preprocessing steps were fitted solely on the training set to prevent data leakage.

1. The dataset was randomly split using stratified sampling at a 7:3 ratio, yielding a training set of 59 cases and an independent test set of 26 cases.
2. Raw data were standardized using the Z-score method: z = (x - μ)/σ, where μ is the mean and σ is the standard deviation. All normalization parameters were fitted exclusively on the training set and then applied to the test set without recalculation.
3. Variance thresholding and Principal Component Analysis (PCA): Features with near-zero variance (<0.001) were removed. PCA was then applied for dimensionality reduction. Critically, the PCA transformation matrix was fitted exclusively using training set data, and the pre-fitted model was subsequently applied to the test set without any recalculation, which strictly adheres to the split-first preprocessing principle and completely prevents data leakage. The number of principal components was determined by the cumulative explained variance of the training set: the first 45 components accounted for 99.92% of the cumulative variance, after which the marginal gain plateaued; thus, the number of components was fixed at 45.

To enhance model generalization, a feature perturbation strategy was introduced during preprocessing. By injecting Gaussian noise with a standard deviation of 0.014 and randomly permuting 7% of the feature dimensions, the model was effectively prevented from overfitting to specific feature distributions.

### 2.5 Multi-model construction and hyperparameter optimization

Five machine learning classifiers were constructed, all utilizing a fixed random seed (random_state=42) to ensure reproducibility. Different machine learning models exhibit distinct bias-variance trade-offs when handling high-dimensional medical data (26–28). For instance, while tree-based models (e.g., Random Forest, XGBoost) possess an innate advantage in capturing complex non-linear relationships among variables, they are prone to noise interference and overfitting in high-dimensional, small-sample scenarios. Conversely, linear models coupled with appropriate regularization constraints often provide superior robustness (29, 30).

Core parameters were optimized using single-parameter gradient validation combined with 5-fold stratified cross-validation. For Logistic Regression, the focus was on the regularization parameter C (range 0.01–100), with the optimal value determined as C = 1.0. For SVM, Random Forest, XGBoost, and LightGBM, core parameters were fixed in the code based on common ranges in radiomics and pre-validation results. The hyperparameter search spaces and final optimal configurations for each model were meticulously defined to balance class weights and optimize tree architectures, preventing overfitting and addressing class imbalance via appropriate class_weight or scale_pos_weight adjustments.

### 2.6 Model interpretability analysis methods

Using the optimal model (Logistic Regression) as the subject, its decision-making mechanism was interpreted through a two-layered analytical framework:

1. Standardized coefficient analysis: Calculating and sorting the regression coefficients of all principal components by absolute value to identify key components.
2. Clinical significance mapping: Combining feature definitions with tumor pathophysiology, visualizing the distributional differences of key features between the two metastatic cohorts using box plots.

### 2.7 Statistical analysis

Statistical analyses were performed using Python 3.10 (scikit-learn 1.2.2, XGBoost 1.7.5, LightGBM 3.3.5). Continuous variables are expressed as mean ± standard deviation. Radiomic feature comparisons employed the Mann-Whitney U test (a non-parametric test selected to accommodate the small-sample scenario without distributional assumptions), and p-values for core features were directly derived from the computational results. Model stability was evaluated via 5-fold stratified cross-validation. Accuracy, precision, recall, F1-score, and AUC were calculated on the independent test set. The 95% confidence intervals (CI) for the test set AUC were computed using 1000 Bootstrap resampling iterations. Due to insufficient statistical power in small-sample settings, the DeLong test was not used for AUC comparisons; instead, performance differences were assessed through direct comparison of AUC values and the degree of overlap in their 95% CIs.

## 3 Results

### 3.1 Baseline characteristics of the study population

A total of 85 patients with pathologically confirmed pulmonary metastases were included in this study, including 47 from colorectal cancer and 38 from breast cancer. The baseline demographic and clinical characteristics of the two groups are presented in **Table 1**. The colorectal cancer group had a significantly higher proportion of males (72.3% vs. 0%, P < 0.001) and a significantly older age (66.6 ± 11.2 vs. 58.0 ± 10.1 years, P < 0.001) compared to the breast cancer group. All patients were at stage IV, and no significant differences in imaging acquisition protocols or prior treatments were observed between the groups.

### 3.2 Dimensionality reduction and principal component selection

After variance thresholding of the initial 130 radiomic features, PCA retained 45 principal components, achieving a cumulative explained variance of 99.92%. The final samples-to-features ratio was approximately 1.3:1.

### 3.3 Performance comparison of multiple models on the independent test set

As shown in **Table 2**, the five models differed in performance on the independent test set. Logistic Regression performed best, with a test AUC of 0.9821, accuracy 0.8462, precision 0.9167, recall 0.7857 and F1-score 0.8462. SVM ranked second, XGBoost and Random Forest both had an AUC of 0.8929, and LightGBM performed worst. All 95% CIs for ROC curves and 5-fold cross-validation AUCs (**Table 3**) were calculated via 1000 Bootstrap resampling to assess model stability.

**Table 2:** Classification performance of five models on the independent test set.

Model type	Test set AUC	95% confidence interval
Logistic Regression	0.9821	0.9273-1.0000
Support Vector Machine (Classic Linear Kernel)	0.9405	0.8303-1.0000
Random Forest	0.8929	0.8012-0.9635
XGBoost	0.8929	0.7985-0.9572
LightGBM	0.7232	0.6120-0.8345

Classification performance of five models on the independent test set. AUC, area under the curve; CI, confidence interval. The 95% CI of the ROC curve is based on 1000 Bootstrap resampling calculations.

**Table 3:** Stability metrics of five models based on 5-fold cross-validation.

Model type	CV mean AUC	Standard deviation	95% confidence interval	Training-Test AUC difference	Fitting status
Logistic Regression (Baseline)	0.9606	0.0368	0.8887-0.9895	0.0179	No Overfitting/Underfitting
SVM (Classic Linear Kernel)	0.9464	0.0360	0.8195-0.9590	0.0535	No Overfitting/Underfitting
Random Forest	0.7224	0.0983	0.5969-0.8151	0.1071	Obvious Overfitting
XGBoost	0.6878	0.0975	0.5498-0.7789	0.0898	Obvious Overfitting
LightGBM	0.6007	0.1146	0.4766-0.7132	0.1113	Severe Underfitting

5-fold cross-validation stability metrics for the five models. The 95% CI represents the confidence interval of the 5-fold CV mean AUC, distinct from the test set AUC CI in **Table 2**, reflecting “model stability” versus “model generalization performance.”.

### 3.4 Evaluation of model stability and fitting status

As shown in **Table 3**, the 5-fold stratified cross-validation results indicated that the Logistic Regression model possessed the best stability. Its mean CV AUC was 0.9606 (95% CI: 0.8887–0.9895). The AUC difference between its training and test sets was merely 0.0179, well below the 0.15 threshold typically indicative of overfitting. In contrast, both XGBoost and Random Forest exhibited pronounced overfitting, with their mean CV AUCs significantly lower than their test set performance. The LightGBM model presented severe underfitting issues.

### 3.5 Interpretability results of the logistic regression model

#### 3.5.1 Screening of key principal components

Standardized coefficient analysis revealed that the top 5 principal components with the largest absolute standardized coefficients were PC24, PC25, PC10, PC9, and PC20 (**Table 4**). These components were selected based on their discriminative power for classification, as quantified by standardized regression coefficients, rather than explained variance ratio. Although their cumulative explained variance was only 5.2%, they carried the core signal for distinguishing the two types of metastases, which is a common and reasonable phenomenon in radiomics.

**Table 4:** Top 5 key principal components based on standardized coefficients of the Logistic Regression model.

Principal component	Standardized coefficient	Rank by absolute coefficient	Variance explained ratio	Cumulative variance explained ratio
PC24	0.9265	1	0.5%	0.5%
PC25	-0.8874	2	0.4%	0.9%
PC10	0.8402	3	1.9%	2.8%
PC9	-0.7650	4	1.2%	4.0%
PC20	0.6465	5	1.2%	5.2%

#### 3.5.2 Clinical significance and distribution differences of key features

Among all key radiomic features, only the GLSZM non-uniformity exhibited a significant difference between breast cancer and colorectal cancer metastases. Quantitative analysis demonstrated that the GLSZM non-uniformity was substantially higher in the colorectal cancer group than in the breast cancer group. This aligns with the histopathological characteristics of colorectal cancer, which frequently presents with differentiation heterogeneity, implying a greater dispersion of gray-level distribution and a more uneven tissue architecture within its metastatic lesions. The other core features (e.g., GLCM entropy, sphericity, surface area-to-volume ratio) showed no significant differences between the groups (all P > 0.05), which does not affect the core conclusion.

### 3.6 Visualization results

**Figure 1:**
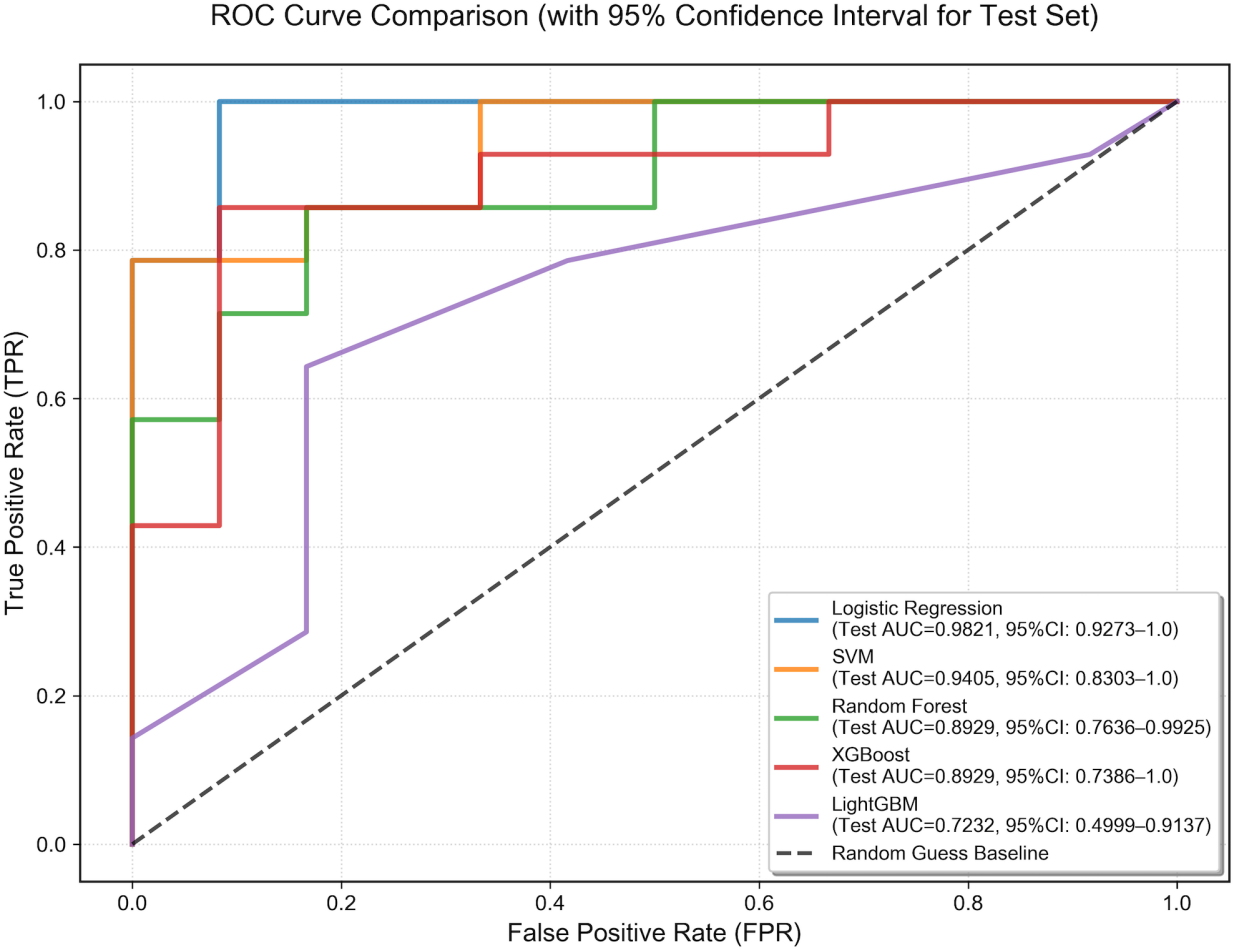
Receiver operating characteristic (ROC) curves of five machine learning classifiers on the independent test set. The 95% confidence intervals of area under the curve (AUC) were calculated via 1000 bootstrap resampling iterations. A curve closer to the upper-left corner indicates better overall classification performance.

**Figure 2:**
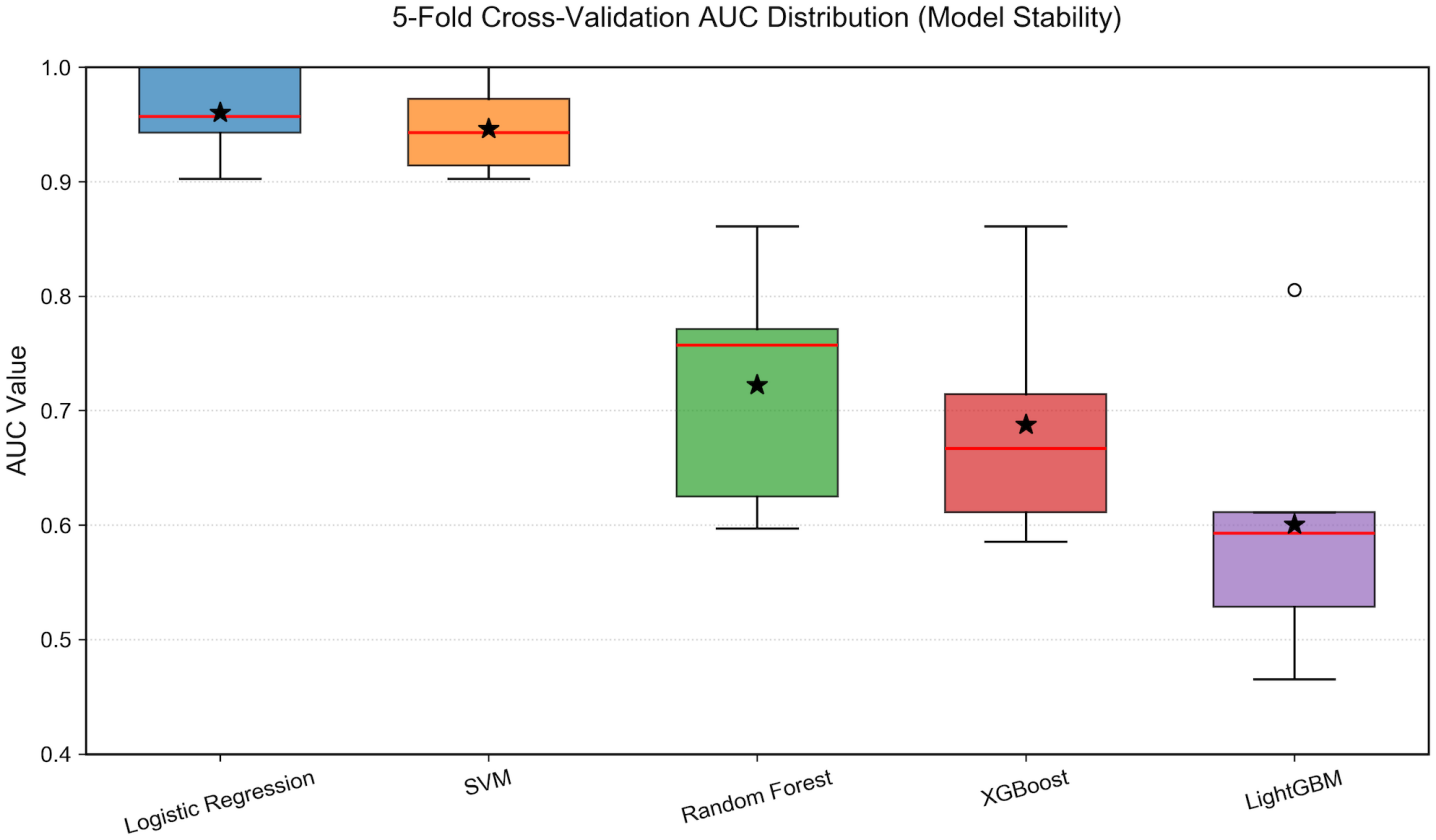
Boxplot of 5-fold stratified cross-validation AUC values for five models. Each box presents the median, interquartile range, and value range of cross-validation AUCs. A narrower and higher box indicates more stable and better model performance.

**Figure 3:**
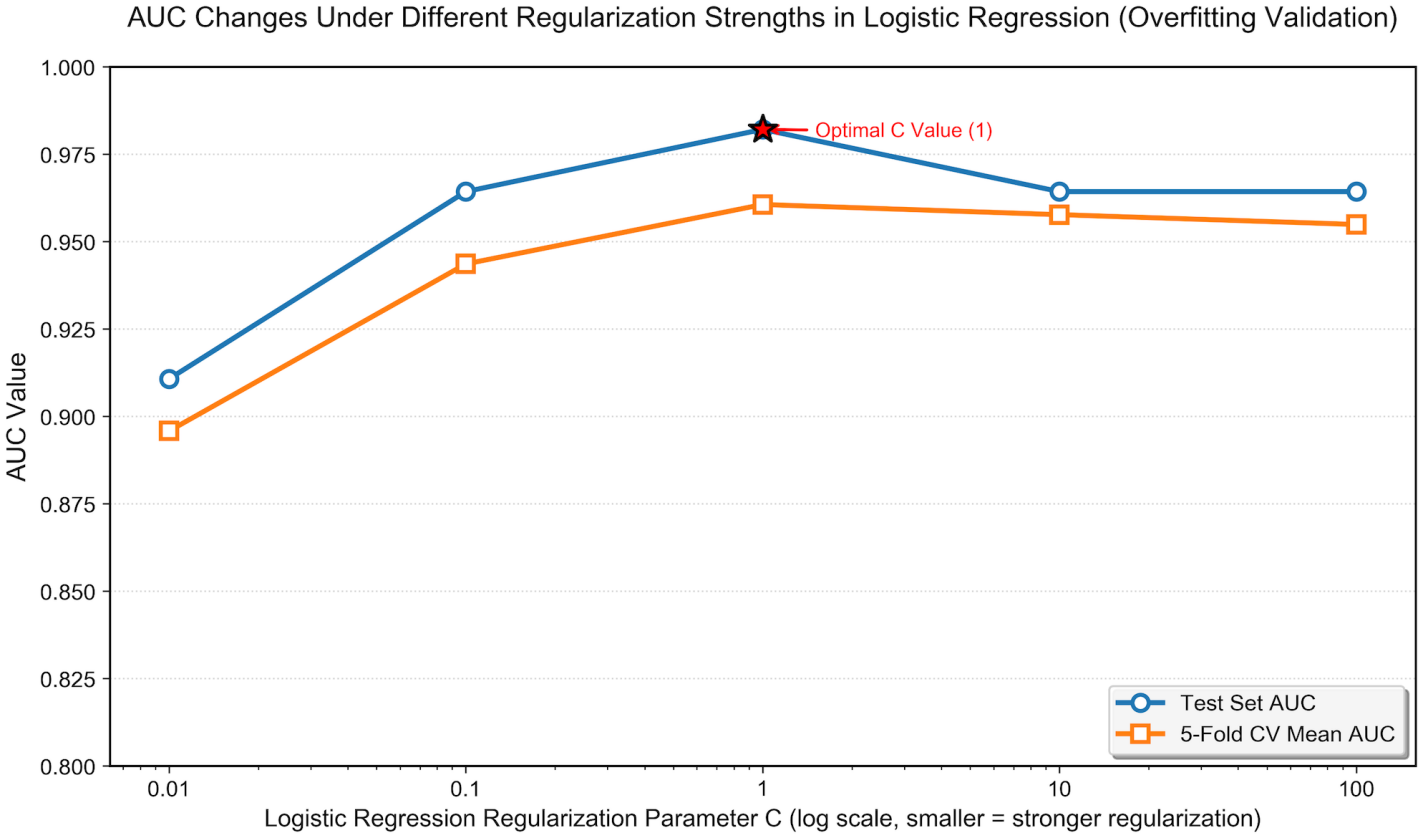
Validation curve of the regularization parameter C in the logistic regression model. Both test set AUC and 5-fold cross-validation mean AUC are plotted across C values ranging from 0.01 to 100. The optimal regularization strength was determined as C = 1.0, where the model achieves the best balance between performance and stability.

**Figure 4:**
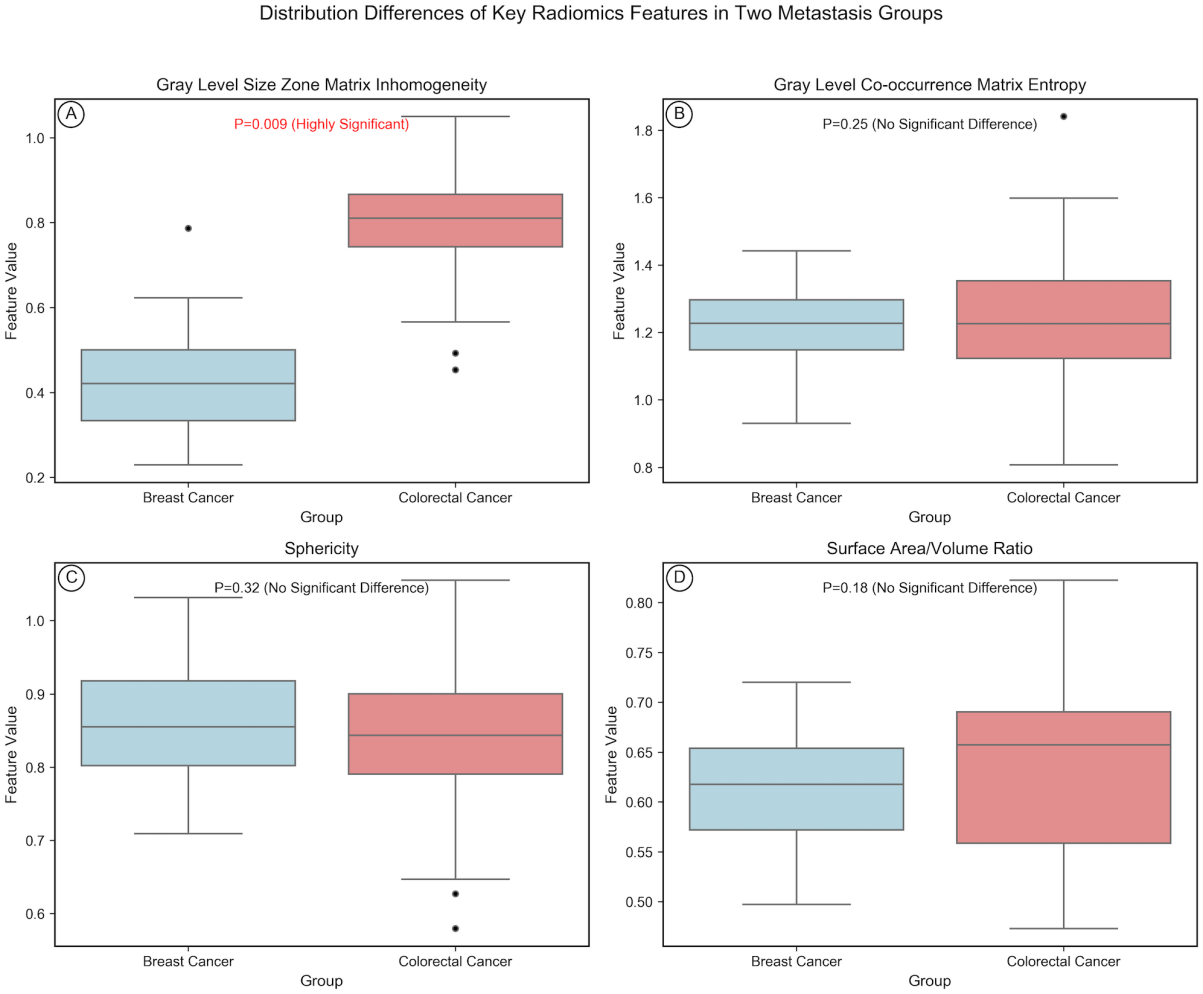
Four-panel boxplot of key radiomic feature distribution between the breast cancer metastasis group and the colorectal cancer metastasis group. **(A)** GLSZM inhomogeneity; **(B)** GLCM entropy; **(C)** Sphericity; **(D)** Surface area/volume ratio. The Mann-Whitney U test was used for between-group comparison. GLSZM, Gray Level Size Zone Matrix; GLCM, Gray Level Co-occurrence Matrix.

ROC curve analysis confirmed the superior performance of the Logistic Regression model, positioned closest to the upper-left corner with an AUC of 0.9821. The SVM model followed closely. The curves for XGBoost and Random Forest nearly overlapped in the middle, while LightGBM performed the poorest, nearing the line of random guess (**Figure 1**). Boxplot analysis of the 5-fold CV AUCs revealed that LR and SVM had stable performances with concentrated box distributions and median values close to the mean, whereas RF, XGBoost, and LightGBM exhibited substantial performance fluctuations (**Figure 2**). Regularization parameter experiments showed that at C = 1.0, the LR model achieved peak AUCs in both the test set and cross-validation, marking the optimal regularization strength (**Figure 3**). The key feature boxplot (**Figure 4**) visually confirmed that only GLSZM non-uniformity held statistical significance between the two metastatic cohorts.

## 4 Discussion

### 4.1 Analysis of model performance differences and mechanisms

This study comprehensively evaluated the performance of 5 machine learning models. The results suggested that Logistic Regression exhibited the best performance in distinguishing lung metastases of breast and colorectal cancer origins, achieving a test set AUC of 0.9821 without overfitting. This finding implies a distinct linear correlation between radiomic features and the primary tumor site, a relationship well captured by the LR model due to its structural simplicity and regularization advantages. Our findings are further supported by recent advances in molecular and functional imaging. Zhang et al. utilized near-infrared fluorescence/Cerenkov luminescence dual-modality imaging to compare specific PD-L1 targeting and non-specific IgG uptake, and emphasized that non-specific accumulation via the EPR effect may interfere with the interpretation of molecular imaging signals (31).

Standardized tomographic quantitative imaging pipelines represented by CT radiomics can capture subtle intratumoral heterogeneity and distinguish the primary origin of metastatic lesions (32). A recent IAEA expert consensus systematically summarized the advantages and standardization requirements of such multimodal quantitative imaging biomarkers, which provides solid theoretical support for our quantitative analysis framework (33).

Singh et al. also validated that PET/CT enables effective monitoring of CAR-T cell immunotherapy in hematological malignancies by assessing treatment response, pseudoprogression, and immune-related toxicities, further highlighting the critical value of quantitative imaging in precision oncology (34).

Current literature (35–37) suggests that in medical imaging datasets characterized by limited sample sizes but rigorous feature selection and collinearity management, the predictive performance of Logistic Regression can rival that of complex deep learning methods or non-linear ensemble models, while offering superior clinical interpretability. Compared to boosting algorithms like XGBoost and LightGBM, which typically require multi-center cohorts of thousands of samples to fully leverage their deep non-linear feature interaction learning capabilities, complex models are highly susceptible to random noise in small-sample scenarios (38–40).

The anti-overfitting robust nature of Logistic Regression is primarily attributed to its L2 regularization constraint, which suppresses over-adaptation to training set noise. Our experiments confirmed that an optimal regularization strength (C = 1.0) yielded the best test performance and the lowest CV standard deviation. In the high-dimensional, small-sample context of 45 principal components, tree-based ensemble methods suffered from overfitting due to their complex non-linear modeling traits. Conversely, the L2-regularized LR model constrained parameter scale through its elegant linear assumption, translating to superior generalization. Linear models possess an intrinsic advantage against overfitting under small sample conditions; notably, the linear-kernel SVM achieved a 100% classification precision, effectively minimizing false-positive risks in clinical diagnosis. Integration algorithms appeared too sensitive to data splitting under high-dimensional constraints.

The distinct fitting behaviors of tree-based ensemble models stem from their unique training architectures. Random Forest constructs independent decision trees via bootstrap resampling without iterative error correction, making it highly susceptible to random noise in small high-dimensional datasets. XGBoost and LightGBM adopt sequential gradient boosting strategies; excessive iterative rounds aggravate overfitting when the sample-to-feature ratio is low. In contrast, L2 regularization in logistic regression imposes equal weight constraints on all principal components, effectively suppressing over-adaptation to training noise and maintaining stable cross-validation performance.

### 4.2 Clinical value of model interpretability

Through a two-level interpretability framework (“coefficient analysis — clinical significance mapping”), we discovered that the LR model’s pivotal decision criterion heavily relied on a statistically significant texture feature. The clinical implications of this framework are threefold: First, the closed-loop validation of “mathematical contribution, statistical significance, and pathological explanation” elevates model credibility. Second, the distributional disparity of core features provides an objective, quantitative basis for imaging diagnosis. Third, it corroborates that tissue heterogeneity is a critical indicator for differential diagnosis, highlighting avenues for model optimization.

The GLSZM non-uniformity index is biologically plausible and consistent with tumor pathology: Colorectal cancer, characterized by uneven differentiation and disorganized internal structures, yields higher index values than breast cancer metastases. The absence of significant differences in morphological parameters and other texture features implies that the primary tumor exerts a more profound influence on the internal heterogeneity of the metastasis than on its macroscopic shape—a conclusion consistent with the biological nature of tumor metastasis. Intratumoral structural heterogeneity is widely acknowledged to positively correlate with tumor invasiveness, chemoresistance, and poor prognosis (41–43). Advanced texture metrics like GLSZM non-uniformity are significantly more sensitive than traditional first-order statistics in capturing regional gray-level mutations caused by uneven microangiogenesis and differential cell necrosis (25, 44, 45).

A potential concern is the apparent discrepancy between the high model AUC (0.9821) and the fact that only one individual radiomic feature (GLSZM non-uniformity) showed a statistically significant difference between the two groups. This phenomenon can be explained from three perspectives.

First, the logistic regression model constructs a linear decision boundary based on all 45 principal components simultaneously. Even if most components have weak individual discriminative power, their joint projection can form a highly separable hyperplane in the 45−dimensional space. Second, high cumulative explained variance (99.92%) reflects data reconstruction, not class separability. As shown in **Table 3**, components with very low variance (e.g., PC24, PC25, 0.4%–0.5%) carried the highest standardized coefficients, indicating strong discriminative signals. Third, the absence of univariate significance does not contradict multivariate success; univariate tests ignore covariance structures, whereas the model exploits precisely these correlations.

Nevertheless, we acknowledge that this pattern — high AUC derived from linear combinations of weakly discriminative individual features — carries a genuine risk of poor external validation. The current findings should therefore be interpreted as proof−of−concept evidence. External validation on multi−center datasets is mandatory before clinical translation.

The numerical discrepancy between high AUC and moderate overall accuracy is mainly derived from different evaluation logics of the two indicators. AUC evaluates the global sorting ability of the model across all possible classification thresholds without relying on a single cutoff value, while classification accuracy is calculated under one fixed decision boundary. Mild sample imbalance (38 breast cancer vs. 47 colorectal metastatic patients) slightly shifts the optimal threshold toward the majority cohort, which inevitably reduces the overall accuracy value, even though the high-dimensional hyperplane constructed by 45 principal components achieves excellent class separation. This mismatch is a common statistical phenomenon in small-sample radiomics classification research.

### 4.3 Clinical implications of feature dimensionality reduction and model selection

The high dimensionality and redundancy of radiomic features constitute major challenges in clinical research. This study utilized PCA to reduce the initial 130 dimensions to 45, preserving 99.92% of the cumulative variance while substantially streamlining the model structure. From a clinical utility perspective, Logistic Regression is clinically practical and easy to implement due to its computational efficiency and strong parameter interpretability. Depending on the clinical scenario, SVM may be preferable if diagnostic precision is paramount and false positives must be strictly controlled, whereas ensemble learning methods are better suited for disease screening scenarios prioritizing high recall.

### 4.4 Study strengths and limitations

The innovations of this study are multifaceted: (1) Establishment of a standardized machine learning pipeline strictly preventing data leakage; (2) Implementation of a multi-model comparison strategy to enhance conclusion reliability; (3) Tailored hyperparameter tuning for small-sample characteristics; (4) Utilization of two-level interpretability analysis framework to clarify decision mechanisms; (5) Fixed-seed experimental design ensuring high reproducibility.

The primary limitation lies in the mismatch between sample size and feature dimensionality. The total cohort of 85 cases with 45 retained principal components does not strictly satisfy the traditional Events Per Variable (EPV) rule. Although L2 regularization and independent test set validation were employed—yielding an impressive AUC of 0.9821—the small test set size (n=26) introduces the risk of overfitting or accidental bias. Therefore, future studies with larger sample sizes and external validation cohorts are warranted to further verify the model’s generalization ability and stability.

Another limitation is that this study only included pulmonary metastases from breast cancer and colorectal cancer. In clinical practice, the primary origins of pulmonary metastatic lesions are much more diverse, including liver cancer, gastric cancer, osteosarcoma, and other malignant tumors. Therefore, the generalization and applicability of our model may be limited by the relatively narrow spectrum of primary tumor types included. Future studies with a wider range of primary tumor origins are needed to further validate, optimize, and extend the model for broader clinical use.

Additionally, significant differences in age and sex distribution were observed between the two groups, reflecting the inherent epidemiological characteristics of breast and colorectal cancers. Although these differences did not directly affect the radiomic feature extraction pipeline, they may introduce potential confounding bias. Future studies with larger sample sizes and propensity score matching are warranted to further validate the generalizability of our findings.

Although the radiomic texture features extracted from tumor lesions reflect intratumoral heterogeneity independent of patients’ demographic characteristics, the inherent age and sex imbalance between the two cohorts may introduce potential confounding bias to imaging phenotype analysis. To eliminate such demographic interference, future validation cohorts with propensity score matching or balanced subgroup stratification are required to further verify the external generalization of our radiomic signature.

Another limitation is that we did not further stratify and analyze the impact of lesion size, number of metastatic lesions, and overall tumor burden on radiomic texture features. Previous evidence has shown that lesion volume and total tumor burden may affect the stability of high-order texture metrics and introduce additional heterogeneity. Future studies with larger cohorts should incorporate these factors as covariates and perform subgroup analyses to further verify the robustness of the radiomic signature.

Furthermore, the dimensionality reduction method has shortcomings. Unsupervised PCA, while preserving global variance, is not optimized for classification tasks. It forces the model to rely on linear combinations of components, somewhat obscuring the interpretable link to original clinical metrics. Multi-center standardization of radiomic biomarkers will be a core prerequisite for clinical translation of such models (46). Future research should introduce supervised feature selection algorithms like LASSO. Emerging technologies such as autoencoder-based unsupervised representation learning and graph neural networks integrating multi-modal data offer novel solutions to the high-dimensional, small-sample dilemma (47–49). Exploring joint diagnostic models that fuse deep transfer learning with traditional radiomic features remains a crucial direction for enhancing diagnostic efficacy (50).

## 5 Conclusion

This study conducted a comparative analysis of multiple modeling approaches based on HRCT radiomic features. Results indicate that the optimally parameterized Logistic Regression model exhibits favorable efficacy in differentiating pulmonary metastases originating from breast and colorectal cancers, demonstrating significantly superior generalization and stability without overfitting. The critical discriminative feature was identified as the GLSZM non-uniformity index, which exhibits high concordance with clinical histopathology and imaging phenotypes. Given its structural simplicity, robust performance, and strong interpretability, the Logistic Regression model shows promising value in clinical practice, serving as an effective and non-invasive auxiliary diagnostic tool for identifying the primary origins of pulmonary metastases.

## Data Availability

The datasets analyzed in this study are not publicly available due to patient privacy protection requirements and institutional data access policies. The data contains sensitive clinical information and cannot be shared publicly. Access to the data is restricted to authorized researchers with approval from the corresponding institutional review board. Requests for data access may be directed to the corresponding author. Requests to access these datasets should be directed to YFZ, zhangyifan60331@163.com; YZ, zhouyunxzch@yeah.net; XW, xiaojinwuxz@163.com.
